# How well do clinical and demographic characteristics predict Patient Health Questionnaire‐9 scores among patients with treatment‐resistant major depressive disorder in a real‐world setting?

**DOI:** 10.1002/brb3.2000

**Published:** 2021-01-05

**Authors:** Jennifer Voelker, Kruti Joshi, Ella Daly, Eros Papademetriou, David Rotter, John J. Sheehan, Harsh Kuvadia, Xing Liu, Anandaroop Dasgupta, Ravi Potluri

**Affiliations:** ^1^ Janssen Scientific Affairs, LLC Titusville NJ USA; ^2^ Janssen Research & Development, LLC Titusville NJ USA; ^3^ SmartAnalyst, Inc New York NY USA; ^4^ Integrated Resources, Inc. Edison NJ USA

**Keywords:** depression, depression severity, Patient Health Questionnaire‐9, treatment‐resistant major depressive disorder

## Abstract

**Objectives:**

To create and validate a model to predict depression symptom severity among patients with treatment‐resistant depression (TRD) using commonly recorded variables within medical claims databases.

**Methods:**

Adults with TRD (here defined as > 2 antidepressant treatments in an episode, suggestive of nonresponse) and ≥ 1 Patient Health Questionnaire (PHQ)‐9 record on or after the index TRD date were identified (2013–2018) in Decision Resource Group's Real World Data Repository, which links an electronic health record database to a medical claims database. A total of 116 clinical/demographic variables were utilized as predictors of the study outcome of depression symptom severity, which was measured by PHQ‐9 total score category (score: 0–9 = none to mild, 10–14 = moderate, 15–27 = moderately severe to severe). A random forest approach was applied to develop and validate the predictive model.

**Results:**

Among 5,356 PHQ‐9 scores in the study population, the mean (standard deviation) PHQ‐9 score was 10.1 (7.2). The model yielded an accuracy of 62.7%. For each predicted depression symptom severity category, the mean observed scores (8.0, 12.2, and 16.2) fell within the appropriate range.

**Conclusions:**

While there is room for improvement in its accuracy, the use of a machine learning tool that predicts depression symptom severity of patients with TRD can potentially have wide population‐level applications. Healthcare systems and payers can build upon this groundwork and use the variables identified and the predictive modeling approach to create an algorithm specific to their population.

## Graphical abstract

In this study, we created and validated a machine learning model using commonly recorded variables within a medical claims database to predict depression symptom severity of patients with TRD based on Patient Health Questionnaire (PHQ)‐9 scores available within an electronic health record (EHR) database. The model was found to predict depression symptom severity category from the three possible severity category choices with an overall accuracy of 62.7%. Of the 116 clinical and demographic variables evaluated in our study, six were found to be the most important predictors. To our knowledge, this is the first study which attempts to predict depression symptom severity of patients with TRD using clinical and demographic characteristics in a medical claims database.

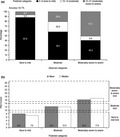

## 1 INTRODUCTION

Major depressive disorder (MDD) is a prevalent chronic mood disorder that affects more than 300 million people globally (World Health Organization, 2017). In the United States, approximately 7.1% (17.3 million) of all adults had at least one major depressive episode in 2017 (National Institute of Mental Health, 2019). The goal of MDD treatment is to achieve complete remission (i.e., full return to baseline functioning with minimal to no residual symptoms; Ballenger, 1999; Trivedi & Daly, 2008; Work Group on Major Depressive Disorder et al., 2010). Pharmacologic treatment with oral antidepressants (ADs) is recommended for patients presenting with mild to moderate symptom severity (Moller et al., 2012; Work Group on Major Depressive Disorder et al., 2010); however, findings from the landmark Sequenced Treatment Alternatives to Relieve Depression (STAR*D) study on the effectiveness of treatment strategies for depression showed that only approximately one‐third (36.8%) of adults with MDD achieved full remission with their first step of AD treatment, and subsequent lines of treatment resulted in substantial decreases in remission rates (approximately 31% and 14% with second and third steps of AD treatment, respectively; Rush et al., 2006).

Treatment‐resistant depression (TRD) is commonly defined as present when a patient with MDD does not reach response or remission after two or more different AD treatments of adequate dose and duration in the current depressive episode (Gaynes et al., 2018). Importantly, over the course of their illness, patients with TRD may experience a wide range of depression symptom severities that span from minimal/no symptoms (i.e., remission) to severe symptoms (American Psychiatric Association [APA], 2013; Kroenke & Spitzer, 2002; Kroenke et al., 2001). Assessment of depression symptom severity may in turn facilitate assessment of critical outcomes for healthcare systems.

Depression symptoms can be assessed using clinician‐administered instruments and/or with patient‐rated instruments, such as the Patient Health Questionnaire (PHQ)‐9. The PHQ‐9 is a self‐reported instrument developed to capture the frequency of nine depression‐related symptoms during the previous two weeks (Kroenke & Spitzer, 2002). Recent guidance by the US Food and Drug Administration has aimed to enhance incorporation of the patient perspective in medical product development and regulatory decision making (Food & Drug Administration, 2019). Moreover, greater communication about patient needs and experiences and the use of patient‐centered care enrich the therapeutic relationship, improve adherence to treatment, and encourage enhanced patient–clinician communication and patient engagement in their care (McNaughton et al., 2019; Peterson et al., 2011).

Unfortunately, neither clinician‐administered nor patient‐rated instruments are generally available in standard medical claims databases nor do these databases include consistent or validated documentation of depression symptom severity; thus, it is difficult to assess the influence of depression symptom severity on a variety of outcomes, including treatment course and response, course of the disease, and other health outcomes. However, some instruments, including the PHQ‐9, are sometimes administered by healthcare providers to patients and recorded in an electronic health record (EHR), providing an opportunity to connect a patient's PHQ‐9 score to their health data from a medical claims database if the two databases can be linked. An effective model that accurately predicts depression symptom severity from commonly recorded variables within medical claims databases could significantly improve understanding of the impacts of the severity of depression symptoms, including its impact on treatment choices made by physicians.

The aim of the current study was to create and validate a model to predict depression symptom severity among patients with TRD using PHQ‐9 scores available within an EHR database linked to a medical claims database.

## 2 METHODS

### 2.1 Data source

This retrospective observational study identified patients between January 2013 and December 2018 (study period) from the Decision Resources Group (DRG) Real World Data Repository (Decision Resources Group). The repository links medical and prescription claims from commercial, Medicaid, and Medicare plans with EHRs to provide longitudinal patient‐level data representative of more than 300 million patients in the United States. Claims data are sourced directly from transactional clearing houses, and EHR data are sourced directly from providers. Both claims and EHR data are linked together by a Health Insurance Portability and Accountability Act—compliant encrypted patient key generated by a third party. PHQ‐9 scores are available for some patients within the EHR database.

### 2.2 Patient identification

Patients were required to have at least one diagnosis code of MDD (*International Classification of Diseases, 9th Revision, Clinical Modification* [ICD‐9‐CM] codes: 296.2x, 296.3x; *International Classification of Diseases, 10th Revision, Clinical Modification* [ICD‐10‐CM] codes: F32.x [excluding 32.8x], F33.x [excluding F33.8]). The index MDD date was defined as the date of the first diagnosis of MDD. Patients were required to have at least 180 days of activity in the database prior to the index MDD date. Patients were then included if they met criteria for TRD during an MDD episode and were ≥ 18 years of age as of the index TRD date (see *MDD and TRD Episode Criteria*). Additionally, patients were required to have at least one PHQ‐9 measurement in the EHR database on or following the index TRD date. Patients with a diagnosis of specific psychiatric disorders (i.e., autism, bipolar disorder, schizophrenia, and other nonmood psychotic disorders) and/or neurologic disorders (i.e., dementia, intellectual disability, traumatic brain injury, Parkinson's disease) during the study period were also excluded.

### 2.3 MDD and TRD episode criteria

As MDD is a chronic, cyclical disorder consisting of distinct time periods of episodes and remission, the following criteria were applied (Figure S1) with the aims to isolate specific episodes of MDD within each patient's longitudinal journey and to identify the incidence of treatment resistance within an episode of MDD.

#### 2.3.1 MDD episode

An MDD episode was defined as a time period that included one or more diagnosis code or treatment for MDD following the first diagnosis code for MDD. Treatments for MDD included oral ADs of adequate dose and duration (≥42 days’ supply of each AD at the minimum dose as recommended by the APA; Work Group on Major Depressive Disorder et al., 2010), and/or procedures used to treat MDD, including electroconvulsive therapy, transcranial magnetic stimulation, and vagus nerve stimulation. An MDD episode was assumed to have started on the date of the MDD diagnosis code, preceded by at least 180 days of a clean period (i.e., without a diagnosis or treatment for MDD), and ended on the date of the last MDD diagnosis code or the end of the days’ supply of an adequate AD medication, whichever came later, followed by at least 180 days of a clean period. The clean period was defined as an absence of MDD diagnosis codes or treatments used for MDD as a means to determine that the patient was in remission of their MDD during this period. Additionally, this allowed for patients to have more than one MDD episode during the study period.

#### 2.3.2 TRD episode

Lines of treatment were evaluated during each MDD episode. The start date of the third line of AD treatment was defined as the index TRD date, based on the assumption that the two previous lines of oral AD treatments of adequate dose and duration had failed to produce a response or remission (see Table S1 for a list of ADs used in determining line of treatment). An AD regimen was considered as failed when the initial AD regimen was augmented with another AD or switched to a new regimen completely. All ADs of adequate dose and duration filled within 30 days of the initial AD claim were considered part of the same regimen.

### 2.4 Variables included in the predictive model of PHQ‐9 scores

A total of 116 clinical and demographic variables typically available in medical claims databases were utilized as predictors associated with depression symptom severity. Variables were identified from (1) a literature search and review of publications, including studies related to causation or association of MDD or depression symptom severity (Amos et al., 2017; APA, 2013; Briggs et al., 2018; Carter et al., 2012; Chin et al., 2016; Gaynes, 2009; Gross et al., 2015; Hinz et al., 2016; Iosifescu et al., 2003; Katzelnick et al., 2011; Mulvahill et al., 2017; Raval et al., 2010; Rossom et al., 2016; Shittu et al., 2014; Wada et al., 2015; Waxmonsky et al., 2012), and (2) discussions with clinicians with expertise in treating patients with TRD. The potential predictors included demographic characteristics, treatment‐specific variables (e.g., site of care, nonpharmacologic treatment, number of prior MDD treatments, specific medications taken for MDD treatment), psychiatric comorbidities, medical comorbidities, measures of healthcare resource utilization, and others (see Table S2 for a full list of variables).

### 2.5 Study outcome

The study outcome of depression symptom severity was measured by PHQ‐9 total score category. The PHQ‐9 total score ranges from 0 to 27 and is typically grouped into six distinct categories ranging from none to severe (Kroenke et al., 2001). For the purpose of this study, the six categories were collapsed into three clinically meaningful categories for the predictive model: none to mild (PHQ‐9 scores, 0–9), moderate (10–14), and moderately severe to severe (15–27). All PHQ‐9 scores recorded on or after the TRD index date in the EHR database were considered for inclusion in the study; each score was treated as a unique outcome, as certain variables may have changed over time and differed between different TRD episodes (e.g., weight, number of previous MDD treatments, comorbidities).

### 2.6 Statistical modeling methodology

A machine learning tool was used because it can test a large number of predictors and identify patterns in the large and heterogeneous dataset used in this study to predict depression symptom severity. A random forest approach was applied to leverage its high prediction accuracy with large numbers of predictors due to the embedded feature selection in the model generation process. The data were randomly divided into training (70%) and validation (30%) datasets, and the random forest classifier, a machine learning technique that enables a large number of weak or weakly correlated classifiers to form a strong classifier (Pal, 2017), was run using the training dataset. After the classifier was trained, the resulting model was applied to the validation dataset in order to provide an unbiased estimate of the model fit. A random forest is a meta‐estimator that fits multiple decision tree classifiers on various subsamples of the dataset and uses averaging to improve the predictive accuracy and limit overfitting. This classifier evolved from and consists of many decision trees. Each uncorrelated decision tree selects a classification of the outcome, and the final choice is based on the aggregated “votes” for each class from the decision tree; the most common classification from the individual trees becomes the result. The input of each tree is sampled data (with replacement) from the original dataset (in this case, the DRG Real World Data Repository). In addition, a subset of features is randomly selected from the optional features to grow the tree at each node. Random forests tend to have high accuracy prediction and can handle large numbers of features due to the embedded feature selection in the model generation process (Pal, 2017). The random forest approach also identifies the rank of importance of predictors by applying a score called the variable importance in projection (VIP; Breiman, 2001), which can be used to identify the most important or influential predictors (the score ranges from zero to one, with a higher score indicating greater importance or influence). While the predictors are ranked, no information on the directionality of the relationship with the outcomes is given by this methodology. Therefore, this study ascertained the direction of effect for selected important variables by calculating the mean value of each by the observed depression symptom severity category. This was done on the entire dataset in a descriptive manner.

Upon completion of the PHQ‐9 classifier, the predicted scores were tested for accuracy against observed PHQ‐9 scores in two ways. First, the overall and individual concordance between the predicted and observed depression symptom severity categories was calculated. Second, in order to verify the use of the three PHQ‐9 depression symptom severity categories (i.e., none to mild, moderate, moderately severe to severe), the mean and median of the observed PHQ‐9 scores within each of the three categories were computed to confirm that the mean and median scores fell within the range for the predicted depression symptom severity category. For example, the mean observed score of a patient predicted to be in the none to mild category should fall within the range (score 0–9) of that category.

## 3 RESULTS

### 3.1 Sample cohort characteristics

In total, 2,077 patients with TRD and 5,356 associated PHQ‐9 measurements were included in the study (Table 1). A total of 116 predictors were included in the model (full list in Table S2) and select variables are reported in Tables 2, 3, 4. The mean age of patients at the time of PHQ‐9 measurement was 51.2 years, 76.9% were female, 52.9% were from the Midwest, and 62.5% had commercial health insurance (Table 2). Anxiety was the most common (41.7%) psychiatric comorbidity within the 180 days prior to the PHQ‐9 measurement, and hypertension was the most common (29.5%) medical comorbidity. Overall, the majority (58.9%) of samples were from patients with one to five psychiatric and/or medical comorbidities.

**Table 1:** Selection of the sample cohort

Step	Label	*N*	% retained from prior step
1	Patients with ≥ 1 MDD diagnosis in the DRG database	5,556,939	
2	*AND* not meeting any specified exclusion diagnoses and having 180 days of preindex MDD activity	3,150,825	56.7
3	*AND* treated with an AD of adequate dose and duration	1,925,108	61.1
4	Number of MDD episodes treated with ≥ 1 AD	2,001,172	
5	*AND* is a TRD episode	224,495	11.2
6	Number of patients represented in Step 5	222,531	
7	Number of patients from Step 6 in the EHR database	110,151	49.5
8	*AND* has ≥ 1 calculated PHQ−9 score after TRD index date	2,168	2.0
9	*AND* is ≥ 18 years of age at TRD index date	2,077	95.8
10	Total PHQ−9 scores from Step 9 patients	5,356	

Note

AD, antidepressant; DRG, Decision Resources Group; EHR, electronic health record; MDD, major depressive disorder; PHQ‐9, Patient Health Questionnaire‐9; TRD, treatment‐resistant depression.

**Table 2:** Clinical and demographic characteristics during the 180‐day period prior to PHQ‐9 measurement^a^

	Total PHQ−9 scores (*N* = 5,356)	
	*n* ^c^	%^c^
Age, years, mean (*SD*)	51.2	16.1
Age‐group, years		
<20	40	0.7
20–29	620	11.6
30–39	699	13.1
40–49	987	18.4
50–59	1,202	22.4
60–69	1,089	20.3
70–79	568	10.6
≥80	151	2.8
Gender		
Male	1,238	23.1
Female	4,118	76.9
Region		
Midwest	2,832	52.9
Northeast	738	13.8
South	1,158	21.6
West	495	9.2
Unknown	133	2.5
Most recent health insurance		
Commercial	3,349	62.5
Medicare	1,045	19.5
Medicaid	837	15.6
Other	125	2.3
Charlson comorbidity index score^d^		
0	3,877	72.4
1	781	14.6
≥2	698	13.0
Most recent severe depression diagnosis code		
Had a severe depression diagnosis code	421	7.9
Days from severe depression diagnosis code, mean (*SD*)	41.7	54.2
Most recent depression diagnosis code		
Mild	312	5.8
Moderate	733	13.7
Severe	295	5.5
Unspecified	1,358	25.4
None	2,658	49.6
Days from most recent depression diagnosis code, mean (*SD*)	29.4	47.7
Count of psychiatric and/or medical comorbidities		
0	1,021	19.1
1–5	3,155	58.9
6–9	890	16.6
≥10	290	5.4
Psychiatric comorbidities		
Any time prior to PHQ−9		
ADHD	470	8.8
180 days prior to PHQ−9^e^		
Anxiety	2,234	41.7
Sleep–wake disorders	1,298	24.2
Nicotine dependence	538	10.0
Psychoactive substance abuse	352	6.6
PTSD	310	5.8
Personality disorder	273	5.1
Alcohol use disorder	192	3.6
Adjustment disorder	104	1.9
OCD	51	1.0
Medical comorbidities		
Any time prior to PHQ−9		
Pulmonary disease (excluding asthma)	977	18.2
Asthma	946	17.7
Heart failure	430	8.0
Cancer	263	4.9
Cerebrovascular disease	241	4.5
Myocardial infarction	176	3.3
Diabetes type 1	169	3.2
Epilepsy	140	2.6
Stroke	121	2.3
180 days prior to PHQ−9^e^		
Hypertension	1,582	29.5
Obesity	1,162	21.7
Dyslipidemia	1,103	20.6
Pain	982	18.3
Diabetes type 2	822	15.3
Ischemic heart disease	667	12.5
Fatigue	585	10.9
Migraine	426	8.0
Fibromyalgia	404	7.5
Nausea	396	7.4
Chronic kidney disease	376	7.0
Nephropathy	372	6.9
Coronary artery disease	311	5.8
Peripheral vascular disease	198	3.7

Abbreviations: ADHD, attention deficit hyperactivity disorder; OCD, obsessive compulsive disorder; PHQ‐9, Patient Health Questionnaire‐9; PTSD, post‐traumatic stress disorder; *SD*, standard deviation.

^a^ Percentages may not add up to 100.0% due to rounding.

^c^ Data are reported as *n* (%) unless otherwise indicated.

^d^ Charlson comorbidity index is designed to predict 1‐year mortality on the basis of a weighted composite score for the following categories: cardiovascular, endocrine, pulmonary, neurologic, renal, hepatic, gastrointestinal, and neoplastic disease.

^e^ Comorbidities reported for < 1% of PHQ‐9 measurements are not shown.

**Table 3:** Health resource utilization

	Total PHQ‐9 scores (*N* = 5,356)	
	*n*	%
Psychotherapy utilization (last 90 days)^a^		
Psychotherapy (any)	1,073	20.0
Psychotherapy, 30 min with patient	552	10.3
Psychotherapy, 45 min with patient	534	10.0
Psychotherapy, 60 min with patient	278	5.2
Psychiatric diagnostic evaluation	175	3.3
Psychiatric diagnostic evaluation with medical	71	1.3
Inpatient/ER utilization (last 90 days)^a^		
Inpatient admission	509	9.5
ER mental health visit	72	1.3
ER visit	681	12.7
Select mental health‐related prescriptions (last 90 days)^a^		
ADs	3,986	74.4
Benzodiazepines or hypnotics	2,048	38.2
Anticonvulsants	1,476	27.6
Antipsychotics	705	13.2
Anxiolytics	449	8.4
Psychostimulants	459	8.6
Any of the above^b^	4,235	79.1
Detailed AD use (last 90 days)^a^		
SSRI	1,770	33.0
DNRI	1,379	25.7
SNRI	1,770	33.0
Serotonin modulator	753	14.1
Norepinephrine–serotonin modulator	355	6.6
Tricyclic/tetracyclic	540	10.1
Most recent physician specialty visit (last 180 days)		
Primary care	2,513	46.9
Psychiatrist	1,264	23.6
None	841	15.7
Other	738	13.8
Prior suicide attempt		
In the last 90 days	14	0.3
In the last 180 days	18	0.3
In the last 365 days	23	0.4

Abbreviations: AD, antidepressant; DNRI, dopamine–norepinephrine reuptake inhibitor; ER, emergency room; PHQ‐9, Patient Health Questionnaire‐9; SNRI, serotonin–norepinephrine reuptake inhibitor; and SSRI, selective serotonin reuptake inhibitor.

^a^ Items reported for < 1% of PHQ‐9 measurements are not shown.

^b^ Including lithium and thyroid medications.

**Table 4:** Summary of PHQ‐9 Score Characteristics^a^

	Total PHQ−9 scores (*N* = 5,356)	
	*n* ^b^	%^b^
PHQ‐9 score, mean (*SD*)	10.1	7.2
PHQ‐9 score category		
None to mild (0–9)	2,686	50.1
Moderate (10–14)	1,120	20.9
Moderately severe to severe (15–27)	1,550	28.9
Days from index TRD, mean (*SD*)	571.4	443.3
Year of PHQ‐9 measurement		
2013	27	0.5
2014	153	2.9
2015	438	8.2
2016	844	15.8
2017	1,842	34.4
2018	2,052	38.3

Abbreviations: PHQ‐9, Patient Health Questionnaire‐9; *SD*, standard deviation; TRD, treatment‐resistant depression.

^a^ Percentages may not add up to 100.0% due to rounding.

^b^ Data are reported as *n* (%) unless otherwise indicated.

Analysis of healthcare resource utilization in the 90 days preceding PHQ‐9 measurement showed that 20.0% of samples were from patients who had a record for psychotherapy, 9.5% from patients who had an all‐cause inpatient visit, and 12.7% from patients who had an all‐cause emergency room visit (Table 3). The majority (74.4%) of samples were from patients who had used an AD in the 90 days preceding PHQ‐9 measurement, and a greater proportion (79.1%) of samples were from patients who had used any of a select group of mental health‐related prescriptions (see Table 3 for list).

The mean (standard deviation) PHQ‐9 score among all samples in the cohort was 10.1 (7.2), indicating moderate depression symptom severity (Table 4). By distribution, it was observed that approximately half (50.1%) of the scores fell in the none to mild category.

### 3.2 Outcomes of the machine learning predictive model

**Figure 1:**
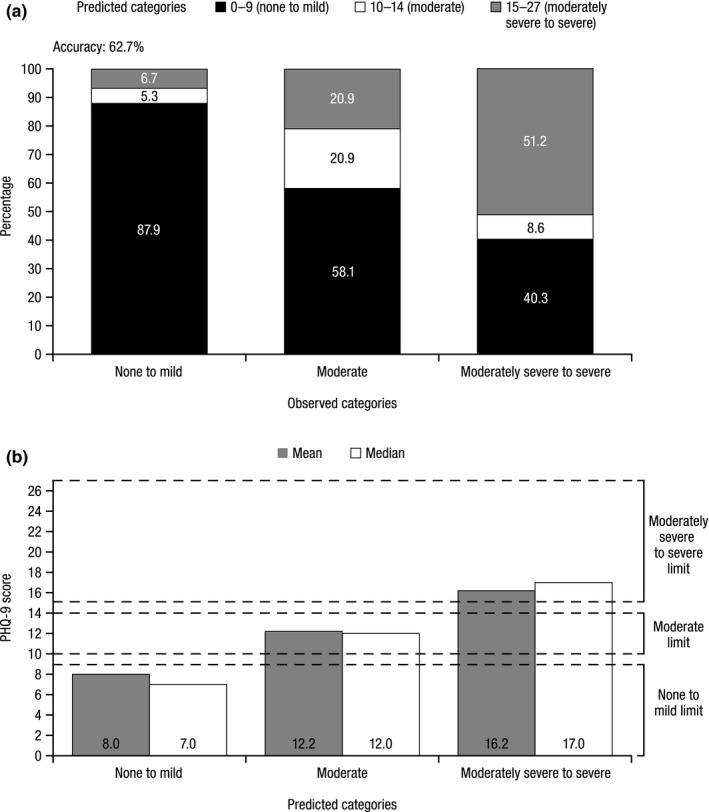
Random forest predictions. (A) Predicted versus observed PHQ‐9 depression symptom severity categories. ^†^(B) Mean and median observed PHQ‐9 scores for each predicted depression symptom severity category. ^†^PHQ‐9, Patient Health Questionnaire‐9.^†^Values are from the 30% validation sample

After training the random forest classifier with the 116 predictors and applying it to the validation dataset, the model yielded predicted PHQ‐9 depression symptom severity categories that corresponded to the correct observed PHQ‐9 categories for 62.7% of patients (Figure 1a). The highest level of concordance between the predicted and observed depression symptom severity categories was found in the none to mild category; 87.9% of those who had observed scores within the none to mild category were accurately predicted to be in the none to mild category. This varied across the other two categories, with the next‐best prediction occurring in the moderately severe to severe category, where 51.2% were accurately predicted. The lowest prediction accuracy occurred in the moderate category, with 20.9% accurately predicted. Furthermore, the mean and median observed PHQ‐9 scores fell within the appropriate range of each predicted depression symptom severity category (mean observed PHQ‐9 score for the predicted none to mild category, 8.0; moderate category, 12.2; moderately severe to severe category, 16.2; Figure 1b).

### 3.3 Important predictors

Out of the 116 predictors included in the random forest classification model, 70 had a VIP score of at least 0.6. Six predictors had a VIP score of at least 0.8 and thus were considered to be the most important predictors in this study. In order of importance, these six predictors were days from index TRD date to PHQ‐9 measurement (VIP, 1.00); days from the last MDD diagnosis with depression classification of severe (0.993); days from the last MDD diagnosis with any known depression classification (0.915); suicide attempt in last 180 days (0.913); days from last MDD diagnosis, with or without known depression classification (0.906); and number of serotonin–norepinephrine reuptake inhibitor (SNRI) prescriptions in the last 90 days (0.843).

**Figure 2:**
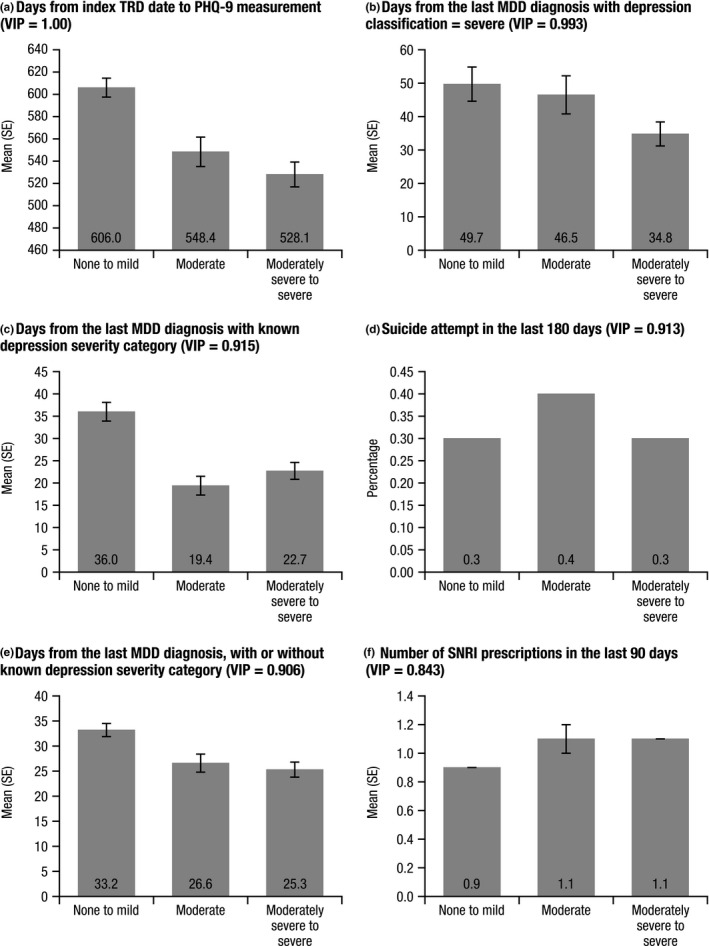
Mean values of the six most important predictors (VIP score ≥ 0.8) by observed depression symptom severity category for all PHQ‐9 scores in the sample cohort. MDD, major depressive disorder; PHQ‐9, Patient Health Questionnaire‐9; SE, standard error; SNRI, serotonin–norepinephrine reuptake inhibitor; TRD, treatment‐resistant depression; and VIP, variable importance in projection

In order to assess the direction of each effect, the mean values of these six predictors were examined by observed depression symptom severity in the 5,356 PHQ‐9 scores in the sample cohort (Figure 2). In general, greater depression symptom severity was associated with a shorter gap from the index TRD date or the last MDD diagnosis to the PHQ‐9 measurement, as well as a higher mean number of SNRI medications in the last 90 days for the moderately to severe and moderate categories compared to the none to mild category (1.1 versus. 1.1 versus. 0.9, respectively).

## 4 DISCUSSION

In a sample of 5,356 PHQ‐9 scores corresponding to 2,077 patients with TRD, this study found using a machine learning predictive model that commonly recorded variables within a medical claims database can be used to predict the depression symptom severity category from the three possible severity category choices with an overall accuracy of 62.7%. While there was variability in the accuracy of the model between the three categories, the observed mean score of patients in each predicted depression category was still within the threshold range of that category. While these results are encouraging, there is yet considerable likelihood of false positives, which is a concern with this model. It is also possible that with the large number of possible predictors used, we overfit our training dataset and this contributed to the results seen with the validation dataset. We hope that these concerns can be alleviated with the help of more advanced machine learning techniques that can improve the accuracy of the model while relying on fewer predictors.

Of the 116 clinical and demographic variables available in medical claims databases, six were found to be the most important predictors in this study. Overall, the findings suggest that the model may be useful to identify important variables researchers should consider when evaluating risk and outcomes across a population with TRD, such as the time from the outcome of interest to the last MDD diagnosis code or index date of TRD.

To our knowledge, this is the first study which attempts to predict depression symptom severity on the PHQ‐9 instrument by using clinical and demographic characteristics among adults with TRD. However, machine learning techniques have been used to predict depression symptom severity in other contexts. One such study validated a previously generated model by using data prospectively collected from individuals with lifetime MDD in two US National Comorbidity Surveys (Kessler et al., 2016; van Loo et al., 2014). Information gathered from the fully structured interview in the first survey was used to predict, among other outcomes, depression symptom severity in the second survey. Severity was based on patient hospitalization for depression, current disability due to depression, and history of suicide attempt. Interestingly, prediction using the machine learning model was better than when using a traditional logistic regression model, even though the former included fewer predictors (10–12 versus 23). In another study, metabolites in the blood plasma of psychiatric patients were associated with patients’ depression symptom severity as measured by the PHQ‐9 and Hamilton Depression Rating Scale (HAMD)‐17 (Setoyama et al., 2016). Five plasma metabolites, among 123 detected, were found to predict depression symptom severity. Further, a machine learning model was developed in this study based on metabolites specifically associated with symptoms of depression including suicidal ideation to predict whether patients had suicidal ideation.

While depression symptom severity classifications are available via ICD codes in some medical claims databases, they are often only recorded for a subset of patients and at a sparse frequency. For example, a recent retrospective database analysis of patients with TRD with up to 2.5 years of follow‐up demonstrated that some medical claims for MDD included ICD codes with severity specifiers; however, not all patients had ICD codes that explicitly classified patients’ depression symptom severity categories (i.e., mild, moderate, severe; Pilon et al., 2019). Additionally, ICD codes for MDD symptom severity are not grounded in either a clinician‐ or patient‐reported validated instrument, and thus rely on subjective assessment of MDD severity. The current study is, to our knowledge, the first to predict depression symptom severity using a validated patient‐reported instrument among adults with TRD using clinical and demographic predictors available in a medical claims database. Notably, demographic correlates of the patient population in this study were consistent with previous national epidemiologic surveys (Hasin et al., 2005, 2018). MDD was more prevalent among women and was associated with other psychiatric disorders, especially generalized anxiety disorder.

Implementation of a predictive model that estimates clinically relevant rating scale scores or categories (e.g., PHQ‐9 score category) can have multiple applications—such as (1) to allow population health decision‐makers with access to claims data that lack PHQ‐9 scores to estimate depression severity among the TRD population they manage and to develop appropriate policies to aid this population, or (2) to address potential confounding due to depression severity in evaluation of comparative effectiveness of TRD treatments in studies where this measure may not be available, or (3) for managed care organizations to compare the imputed PHQ‐9 scores among their population of patients that initiated a treatment to ascertain if depression severity potentially played a role in treatment choices by their physicians. It bears clarification that this predictive model has not been designed to be used to estimate depression severity for individual patients prospectively with a view to influencing any clinical or treatment‐related decision by clinicians.

Other predictive models in depression have been developed to identify predictors of remission and response to therapy. In one study, predictors of remission were identified based on placebo‐treated patients with MDD in double‐blind randomized clinical trials (Nelson et al., 2012). Four predictors were identified: less severe depression symptoms, younger age, less anxiety, and shorter current MDD episode duration. Interestingly, anxiety was not identified as a predictor of depression symptom severity in the current study, notwithstanding the high proportion of the study population (41.7%) with comorbid anxiety disorder. In another study, predictors of response and remission among inpatients with depression were identified (Riedel et al., 2011). Common predictors for both outcomes were fewer previous hospitalizations and episode duration less than 24 months. Of note, the presence of suicidality was found to be a predictor of response. While this seems counterintuitive, the authors speculated that suicidality served as a surrogate indicator of depression symptom severity and, as “response” was defined as a percentage reduction in HAMD‐21 score from initial to final visit, this outcome may be inherently biased in favor of patients with higher HAMD‐21 scores, and thus more severe depression, at baseline. Indeed, in the current study, suicide attempt within the 180 days prior to PHQ‐9 measurement was among the most important predictors of depression symptom severity, but the association was complex in that higher suicidality was found to be associated with moderate depression symptom severity. Moreover, while the current study identified other important predictors of depression symptom severity, due to the different nature of the design, to our knowledge, no other studies in the literature have reported similar findings related to the number of SNRI prescriptions or the time from certain events to depression symptom assessment.

Strengths of this study include that the PHQ‐9 is a validated and widely used instrument to assess depression symptoms, as opposed to using ICD categories which have not been validated. In this study, depression symptom severity was predicted among patients with TRD using a machine learning approach from a large, linked database containing more than 5,000 PHQ‐9 values. This analysis used a test/validation set and commonly available variables, making the results practical for other users. This study also identified the most important variables of practical value to other researchers selecting appropriate variables to adjust for during retrospective database analyses of TRD patients, but unable to build a full imputation model for PHQ‐9 scores.

This study has several limitations. First, the predictive capabilities and accuracy of the random forest classifier are limited to the predictors chosen for the study, as well as the study's definitions of TRD and each depressive episode, which impact confirmation of TRD, patients’ classification, and study findings. The large number of predictors used may have caused an overfitting of the training dataset, particularly in the context of a database different from the one used for the study. There was a large average gap (571 days) observed between the index TRD date and PHQ‐9 measurement date. Furthermore, the study population may not be representative of other populations (e.g., patients without PHQ‐9 data in claims databases may have a different presentation). The analysis does not capture the underreporting of clinical characteristics not covered by payers (e.g., psychotherapy, outpatient visits with psychiatrists). Within the context of these limitations, the real‐world validation study reported here identified several readily accessible baseline patient variables that appear to predict depression symptom severity with high accuracy.

In conclusion, while acknowledging the substantial room for improvement in accuracy, the use of a machine learning tool that predicts depression symptom severity of patients with TRD with the help of commonly available variables in a medical claims database can potentially have wide population‐level applications. Healthcare systems and payers can build upon this groundwork and use the variables identified and the predictive modeling approach to create an algorithm specific to their population, leading to ultimately, provision of better care and improved health outcomes for this vulnerable population.

## CONFLICTS OF INTEREST

J. Voelker, K. Joshi, E. Daly, and J.J. Sheehan are employees of Janssen, and may be stockholders in Johnson & Johnson. E. Papademetriou, D. Rotter, X. Liu, A. Dasgupta, and R. Potluri are affiliated with SmartAnalyst, Inc. H. Kuvadia is an employee of Integrated Resources, Inc., providing services for Janssen Scientific Affairs, LLC.

## AUTHOR CONTRIBUTIONS

J. Voelker, K. Joshi, H. Kuvadia, and R. Potluri contributed to the conception/design of the study, and analysis and interpretation of the data. E. Daly contributed to the conception/design of the study and interpretation of the data. E. Papademetriou and D. Rotter contributed to the acquisition, analysis, and interpretation of the data. J.J. Sheehan contributed to the conception/design of the study, and acquisition and interpretation of the data. X. Liu contributed to the analysis of the data. Anandaroop Dasgupta contributed to the analysis and interpretation of the data. All authors contributed to drafting/critical review of the manuscript and approved the final version.

## ETHICAL STATEMENT

All patient data contained with the DRG database are de‐identified and in compliance with the Health Insurance Portability and Accountability Act. Thus, no Institutional Review Board approval was required for this study.

## Supporting information

Fig S1

Supplementary Material

## Data Availability

The data that support the findings of this study are available from Decision Resources Group (DRG) but restrictions apply to the availability of these data, which were used under license for the current study, and so are not publicly available. More information about accessing the DRG Real World Data Repository can be found at: https://decisionresourcesgroup.com/solutions/real‐world‐data/.
