# Supplementary material for: How well do clinical and demographic characteristics predict Patient Health Questionnaire‐9 scores among patients with treatment‐resistant major depressive disorder in a real‐world setting?
Source: Brain Behav. 2021 Jan 5;11(2):e02000. doi: 10.1002/brb3.2000 (PMC7882175; doi:10.1002/brb3.2000)
Supplement: Supplementary file 2 — Supplementary Material [file BRB3-11-e02000-s002.docx]

**Supplemental Materials**

**Table S1. List of ADs^†^**

| **AD** | **Minimum daily dose** |
| --- | --- |
| SSRIs |  |
| Citalopram | 20 mg |
| Escitalopram | 10 mg |
| Fluoxetine | 20 mg |
| Fluvoxamine | 50 mg |
| Fluvoxamine, extended release | 100 mg |
| Paroxetine | 20 mg |
| Paroxetine, extended release | 12.5 mg |
| Sertraline | 50 mg |
| DNRI |  |
| Bupropion | 150 mg |
| SNRIs |  |
| Desvenlafaxine | 50 mg |
| Duloxetine | 60 mg |
| Levomilnacipran | 20 mg |
| Milnacipran | 12.5 mg |
| Venlafaxine | 37.5 mg |
| Serotonin modulators |  |
| Nefazodone | 50 mg |
| Trazodone | 150 mg |
| Vilazodone | 10 mg |
| Vortioxetine | 10 mg |
| Norepinephrine-serotonin modulator |  |
| Mirtazapine | 15 mg |
| Tricyclics and tetracyclics |  |
| Amitriptyline | 25 mg |
| Amoxapine | 50 mg |
| Clomipramine | 25 mg |
| Desipramine | 25 mg |
| Doxepin | 25 mg |
| Imipramine | 25 mg |
| Maprotiline | 75 mg |
| Nortriptyline | 25 mg |
| Protriptyline | 10 mg |
| Trimipramine | 25 mg |
| MAOIs |  |
| Isocarboxazid | 10 mg |
| Phenelzine | 15 mg |
| Selegiline | 6 mg |
| Tranylcypromine | 10 mg |

AD, antidepressant; APA, American Psychiatric Association; DNRI, dopamine-norepinephrine reuptake inhibitor; MAOI, monoamine oxidase inhibitor; SNRI, serotonin-norepinephrine reuptake inhibitor; SSRI, selective serotonin reuptake inhibitor; TRD, treatment-resistant depression.

^†^Starting doses were based on the recommended starting dose indicated by the APA ([APA](#_ENREF_1), 2010). The starting doses of antidepressants not included in the APA guidelines were based on the starting dose indicated in the label. Other selected medications included antidepressant-antipsychotic combination treatments indicated for TRD.

**Table S2. Variables for Inclusion in the Predictive Model**

|  | **Any time prior to PHQ-9 measurement** | **90 days prior to PHQ-9 measurement** | **180 days prior to PHQ-9 measurement** |
| --- | --- | --- | --- |
| **Demographic characteristics** | Age at PHQ-9 (continuous) |  | Charlson comorbidity index (continuous) |
|  | Age at PHQ-9 (categorical) |  | Charlson comorbidity index (categorical) |
|  | Age at TRD (continuous) |  | Medicaid claim |
|  | Gender |  | Most recent weight value |
|  | Most recent insurance status |  |  |
|  | Region |  |  |
| **Treatment-specific variables** | Count of comorbidities (continuous) |  | MDD severity Dx |
|  | Count of comorbidities (categories) |  | Most recent site of care |
|  | Days from latest MDD Dx with severity categorisation |  | Prior MDD Dx |
|  | Days from latest severe MDD Dx to PHQ-9 with severity classification = severe |  | Severe depression Dx |
|  | Days from MDD Dx to PHQ-9 |  | Physician specialty |
|  | Days from TRD index date |  |  |
|  | Num of prior MDD episodes (including current) |  |  |
|  | Year of PHQ-9 |  |  |
| **Psychiatric comorbidities** | ADHD | Opioid dependence | Adjustment disorder |
|  |  | Suicide attempt | Alcohol use disorder |
|  |  |  | Anxiety |
|  |  |  | Eating disorder |
|  |  |  | Nicotine dependence |
|  |  |  | OCD |
|  |  |  | Personality disorder |
|  |  |  | PTSD |
|  |  |  | Psychoactive substance abuse |
|  |  |  | Sleep-wake disorders |
|  |  |  | Suicide attempt |
|  |  |  | Tourette’s disorder |
|  |  |  | **365 days prior to PHQ-9 measurement** |
|  |  |  | Suicide attempt |
| **Medical comorbidities** | Asthma |  | Chronic kidney disease |
|  | Cancer |  | Coronary artery disease |
|  | Cerebrovascular disease |  | Diabetes type 2 |
|  | Diabetes type 1 |  | Dyslipidaemia |
|  | Epilepsy |  | Erectile dysfunction |
|  | Heart failure |  | Fatigue |
|  | Myocardial infarction |  | Fibromyalgia |
|  | Pulmonary disease (excluding asthma) |  | Hypertension |
|  | Stroke |  | Ischemic heart disease |
|  |  |  | Migraine |
|  |  |  | Nausea |
|  |  |  | Nephropathy |
|  |  |  | Obesity |
|  |  |  | Pain |
|  |  |  | Peripheral nerve disease |
|  |  |  | Peripheral vascular disease |
|  |  |  | Pregnancy |
|  |  |  | Sexually transmitted diseases^†^ |
| **Measures of health care resource utilisation** |  | Anticonvulsant Rx | Nonpharmacologic treatment |
|  |  | ADs Rx | Rx for insulin use |
|  |  | Antipsychotic Rx |  |
|  |  | Anxiolytic Rx |  |
|  |  | Any mental health–related Rx |  |
|  |  | Benzodiazepine Rx |  |
|  |  | Drugs used in alcohol dependence |  |
|  |  | ER mental health visit |  |
|  |  | ER visit |  |
|  |  | Group psychotherapy |  |
|  |  | Hypnotic Rx |  |
|  |  | Inpatient admission |  |
|  |  | Lithium Rx |  |
|  |  | MAOI Rx |  |
|  |  | Mental health admission |  |
|  |  | DNRI Rx |  |
|  |  | Norepinephrine-serotonin modulator Rx |  |
|  |  | Num anticonvulsant Rx |  |
|  |  | Num ADs Rx |  |
|  |  | Num antipsychotic Rx |  |
|  |  | Num anxiolytic Rx |  |
|  |  | Num any MDD Rx |  |
|  |  | Num lithium Rx |  |
|  |  | Num MAOI Rx |  |
|  |  | Num DNRI Rx |  |
|  |  | Num norepinephrine-serotonin modulator Rx |  |
|  |  | Num of ER mental health visits |  |
|  |  | Num of ER visits |  |
|  |  | Num of inpatient days |  |
|  |  | Num of mental health admissions |  |
|  |  | Num psychostimulant Rx |  |
|  |  | Num serotonin modulators Rx |  |
|  |  | Num SNRI Rx |  |
|  |  | Num SSRI Rx |  |
|  |  | Num thyroid Rx |  |
|  |  | Num tricyclics/tetracyclics Rx |  |
|  |  | Psychostimulant Rx |  |
|  |  | Psychotherapy |  |
|  |  | Psychotherapy, 30 minutes with patient |  |
|  |  | Psychotherapy, 45 minutes with patient |  |
|  |  | Psychotherapy, 60 minutes with patient |  |
|  |  | Psychotherapy diagnostic evaluation |  |
|  |  | Psychotherapy diagnostic evaluation with medical |  |
|  |  | Serotonin modulators Rx |  |
|  |  | SNRI Rx |  |
|  |  | SSRI Rx |  |
|  |  | Thyroid Rx |  |
|  |  | Tricyclics/tetracyclics Rx |  |

AD, antidepressant; ADHD, attention deficit hyperactivity disorder; DNRI, dopamine-norepinephrine reuptake inhibitor; Dx, diagnosis; ER, emergency room; HIV, human immunodeficiency virus; MAOI, monoamine oxidase inhibitor; MDD, major depressive disorder; num, number; OCD, obsessive compulsive disorder; PHQ-9, Patient Health Questionnaire–9; PTSD, post-traumatic stress disorder; Rx, prescription; SNRI, serotonin-norepinephrine reuptake inhibitor; SSRI, selective serotonin reuptake inhibitor; TRD, treatment-resistant depression.

^†^The HIV variable (any time prior to PHQ-9 measurement) was integrated with the sexually transmitted disease variable. There was no overlap between HIV and other sexually transmitted disease cases.

**Figure S1. Identification of patients with TRD.**


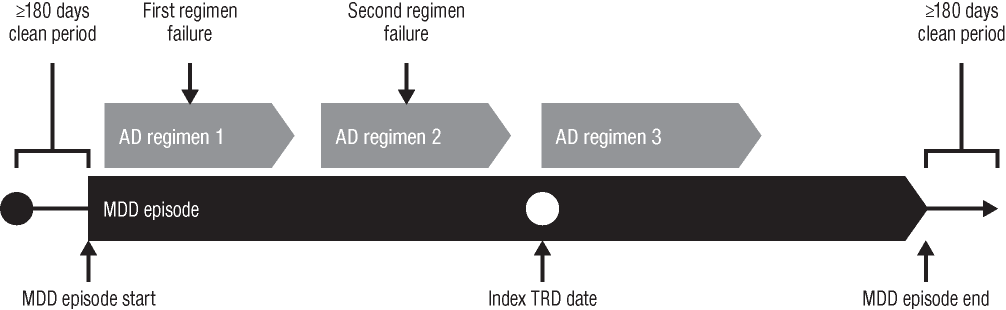


AD, antidepressant; MDD, major depressive disorder; TRD, treatment-resistant depression.

**Reference**

APA. (2010). Practice guideline for the treatment of patients with major depressive disorder (3rd ed.). American Psychiatric Association.
